# COMPAC: COMputable Phenotype for Asthma in Children

**DOI:** 10.21203/rs.3.rs-6370010/v1

**Published:** 2025-05-09

**Authors:** Jennifer Fishe, Jinqian Pan, David Fedele, Mengxian Lyu, Morgan Henson, Nolan Menze, Taylor Scott, Taylor Munson, Meghan Carberry, Abigail Dyer, Kelli Ketola, Yonghui Wu, Jie Xu

**Affiliations:** University of Florida

**Keywords:** Computable Phenotype, Electronic Health Record, Pediatric Asthma

## Abstract

**Background:**

Pediatric asthma is one of the most common chronic diseases of childhood. Reliable identification of pediatric asthma patients in electronic health records (EHRs) is essential for both research and clinical care. However, existing computable phenotypes (CPs) exhibit varying effectiveness. This study aims to evaluate current CPs and develop a new CP, named COMPAC (COMputable Phenotype for Asthma in Children), to improve EHR-based identification of pediatric asthma patients.

**Methods:**

Multiple CP rules were designed using various combinations of diagnosis codes, prescriptions, and clinical note text. A cohort from the University of Florida Integrated Data Repository (IDR) was used for validation through manual chart reviews. Performance was assessed using standard metrics and compared to existing CPs. Additionally, bootstrapping and demographic subgroup analyses were conducted to compare the performance of the new COMPAC to previously published CPs.

**Results:**

COMPAC demonstrated improved case identification compared to existing CPs, with high sensitivity (0.728; 95% confidence interval [CI]: 0.607–0.864), positive predictive value (0.886; 95% CI: 0.737–1.0), and an overall F1 score of 0.797 (95% CI: 0.682–0.90). Notably, COMPAC outperformed two previously published CPs in terms of F1 score. Performance varied across demographic subgroups, with COMPAC showing the best results in males, non-Hispanic Whites, and the 6–12 year-old age group, though its performance was lower in the 2–5 year-old age range.

**Conclusion:**

COMPAC offers an improved approach for pediatric asthma case identification in EHRs. However, further validation across different sites and refinement to capture a broader range of clinical presentations are necessary to optimize its sensitivity and specificity.

## 1. BACKGROUND

Pediatric asthma is one of the most common chronic diseases of childhood, affecting an estimated 4.5 million children in the United States (US). ^1^ Childhood asthma is a major source of morbidity, resulting in an annual estimated 270,000-500,000 emergency department visits and 27,000-64,000 hospitalizations in the US, ^2,3^ as well as over 10 million missed school days ^4^ and $5.92 billion in healthcare costs. ^5^

To mitigate those detrimental effects, pediatric asthma is the focus of intensive research efforts. Furthermore, pediatric asthma research using real-world data (RWD) collected from electronic health records (EHR) holds great promise for generating externally valid, broadly generalizable results. Large amounts of data from heterogeneous patient populations is particularly important for efforts to define clinical sub-phenotypes (“subtypes”) of pediatric asthma that could guide personalized treatments, resulting in improved symptoms and better long-term prognoses.^6^

However, research using retrospective EHR data must accurately identify children with asthma. As asthma is a heterogeneous, evolving, chronic disease, identification of children with asthma is nuanced. The classic “gold standard” for diagnosis of asthma is pulmonary function testing (PFTs),^7^ however PFTs are not commonly done in routine clinical practice, and PFT data can be difficult to locate in the EHR.^8^ Additionally, using diagnosis codes, such as the International Classification of Diseases (ICD), may miss younger children with asthma whom clinicians are reticent to diagnose, or may falsely label someone as having asthma when their asthma has resolved.^9^

Given those challenges, application of a computable phenotype to accurately identify pediatric asthma patients is appropriate. Computable phenotypes (CPs) are reproducible ways of identifying patients of interest from data, and can be rule-based or machine learning-based.^10^ Upon review of the literature, there are four existing pediatric asthma CPs developed for application to longitudinal EHR data. However, those CPs show variable performance and have not been tested at a large scale.

CAPriCORN is a rule-based CP that utilizes structured data, and when originally tested across multiple institutions in the same geographic area had a 96.7% accuracy.^11^ However, when CAPriCORN was applied to two large geographically distinct areas across the United States, its harmonized F1 score was 90.4%.^12^ PheKB is a rule-based CP that utilizes structured and unstructured data. When applied in the same geographically distinct nationwide analysis as CAPriCORN, PheKB had a harmonized F1 score of 75.4%.^13^ NLP-PAC is a CP with a rule-based and machine learning-based version that uses structured and unstructured data. The original publication of NLP-PAC tested its performance at a single institution and found a F1 score of 82.4% for the rule-based version and 86.3% for the machine learning-based version.^14^ When the rule-based NLP-PAC was applied at an external institution, the F1 score was not reported but its sensitivity was 92% and specificity was 96%.^15^ NLP-API is a rule-based CP utilizing structured and unstructured data, and when tested at a single institution had a sensitivity of 86% and specificity of 98%, with no F1 score reported.^16^

Given that broad range of performance, and that there is no consensus pediatric asthma CP, the objective of this study was to test existing pediatric asthma CPs with publicly available code on a large, external RWD source. We also sought to develop a new pediatric asthma CP utilizing combinations of rules from existing CPs. The underlying purpose of our CP development efforts is to accurately identify pediatric asthma patients for a larger project aimed at understanding pediatric asthma subtypes.^6^

## 2. METHODS

### 2.1 Data Source and Regulatory Approval

This study utilized data from the University of Florida (UF) Health Integrated Data Repository (IDR), a large-scale and robust database designed to aggregate, store, and report comprehensive information from the UF Health system’s clinical and research enterprises (i.e., Epic EHR system; Janesville, WI). The UF Health IDR covers a diverse population of over 2 million patients and includes encounters, patient demographics, diagnostic codes, medication records, and unstructured clinical notes, facilitating the identification of pediatric asthma patients. This study was approved by the University of Florida Institutional Review Board (UF IRB#202002779).

### 2.2 Overall Study Design

#### De novo CP development

**Figure 1:**
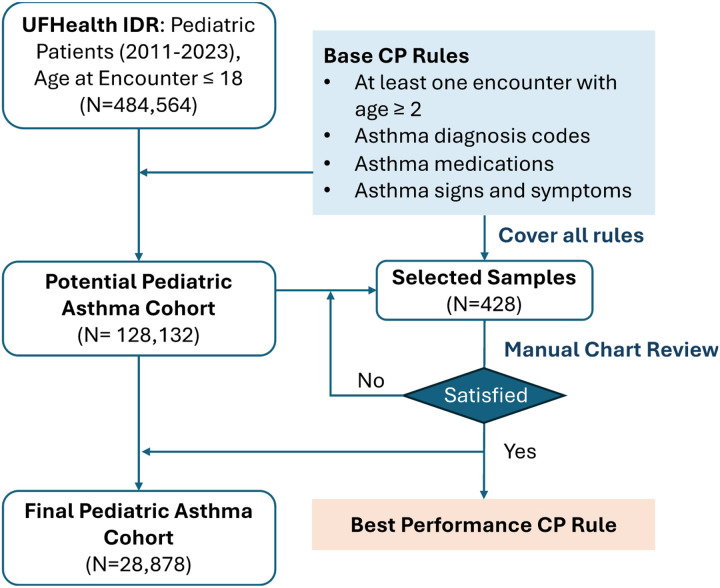
Flowchart of the pediatric asthma CP development process.

We developed a CP to identify pediatric asthma patients through structured and unstructured EHR data. The flowchart of the CP development process is illustrated in Fig. 1. We defined the pediatric asthma age range as > 2 years to avoid confounding with viral-induced wheezing such as bronchiolitis, and < 18 years to exclude adults. However, in our baseline CP rules described below, we included data from when patients were ages of 1-2 years if there was an occurrence of a baseline CP rule during that time and also after the age of 2 years.

### 2.3 Manual chart reviews of selected samples from the potential asthma cohort

Drawing on insights from prior studies^9,11-16^ regarding case-finding algorithms for asthma, and input from pediatric asthma clinical specialists (J.N.F. and D.F.), we established the following initial base rules: (1) patients aged between 2 and 18 years, (2) having at least one asthma diagnosis codes, (3) use of at least two inhaled bronchodilator (BDR) medications, (4) use of at least one inhaled corticosteroid medication, (5) presence of at least one asthma-related sign or symptom (kw1), and (6) presence of at least two asthma-related signs or symptoms (kw2) documented in clinical notes. The relevant value sets, codes, and keywords for these base rules are outlined in Table 1. We then extracted a cohort of patients who met various combinations of those six base rules, as shown in Table 2.

We generated distinct combinations of CP rules by combining the base rules with additional human-driven adjustments based on clinical insights gleaned during clinical note annotations. We first randomly selected 500 patients from the entire cohort. We then assessed whether the selected patients met the CP rules for at least 10 patients. For any CP rule that did not meet this threshold, we randomly selected additional patients from the subset identified by that rule, proportionate to the percentage of randomly selected patients in relation to the whole cohort.

We conducted multiple rounds to ensure that, for the groups meeting the criteria, the proportion of patients identified by each base rule—where only the explicitly mentioned rules are considered, and any unmentioned rule must be absent—among the total number of patients covered by that base rule—where unmentioned rules are ignored and can be either present or absent—was at least 10% higher. This threshold was set to minimize the risk of missing relevant cases. We ensured that at least 18 patients were reviewed for each qualifying group. We chose the 10% threshold to balance two key considerations: (1) ensuring a reasonable number of patients were included for manual chart review, providing sufficient data to validate the rule, and (2) capturing a diverse range of scenarios to avoid overlooking potential phenotypic variations. This iterative process helped refine the CP rules while maintaining both feasibility and comprehensiveness in case identification.

For all the patients within the cohort, we collected their EHR data, including structured data (e.g., demographics, diagnoses, medications) and unstructured clinical notes (e.g., primary care clinic notes, specialty visits, ED and hospital visits, progress notes, discharge summaries, all identified via regular expressions).

To ensure consistency in applying the review criteria across all reviewers, we developed a comprehensive annotation guideline. J.N.F. led the annotation team in reviewing the first 20 selected patients, ensuring adherence to these guidelines. During this initial training phase, all chart reviewers independently evaluated the same 20 samples, with debriefing of the rating of all samples led by J.N.F. Following that initial training review, each selected sample was assigned to two annotators, with a total of four teams of two annotators. In cases of disagreement between the reviewers, the entire study team engaged in discussions to resolve conflicts and reach a consensus. The annotation guideline was iteratively revised based on these discussions to improve clarity and consistency. For the subsets of CP rule combinations that did not achieve satisfactory agreement or were not covered, we selected additional patients for chart review in the later rounds.

### 2.4 CP evaluation

We evaluated the performance of each rule combination using multiple metrics, including sensitivity, positive predictive value (PPV), and F1-score. The final best-performing CP rule was applied to define the pediatric asthma cohort for further analysis. To benchmark our CP, we compared it with two previously established phenotypes: CAPriCORN and PheKB. To adapt the CAPriCORN approach to our current methodology, we modified their criteria, which initially required a diagnosis at the first visit followed by either a diagnosis or prescription at a second visit. We simplified this by requiring either two diagnoses or a combination of diagnosis and prescription. For PheKB, their criteria specify at least three separate visits within one year with mentions of “wheezing” or “asthma.” To align with our approach, we removed the one-year timeframe restriction and only required three separate mentions of “wheezing” or “asthma” across the timeline, regardless of when they appeared. For NLP-PAC and NLP-API, we were unable to obtain source code in order to apply those CPs.

To enhance the evaluation, we conducted bootstrapping on the chart-reviewed patients. For each rule combination, we randomly selected 20 patients identified by the rule from the chart-reviewed cohort using sampling with replacement, and 20 additional patients from the remaining pool of patients, also selected randomly with replacement. This process was repeated for 10,000 iterations. For each rule combination, we reported the mean and 95% confidence intervals for the evaluation metrics.

### 2.5 Demographic subgroup analysis

To evaluate potential performance variability of the developed computable phenotypes (CPs) across different demographic subgroups, we assessed the performance of both the baseline CP and the best-performing CPs within the UF-site chart-reviewed cohort. The subgroups analyzed included gender (i.e., Female, Male), age (i.e., 2–5 years, 6–12 years, and 13–18 years), and race/ethnicity (i.e., Hispanic, Non-Hispanic White, and Non-Hispanic Black). We reported sensitivity, PPV, and F1-score to provide a comprehensive view of the CP’s ability to accurately identify asthma cases (sensitivity and PPV), as well as the balance of the CP’s performance (F1-score).

First, we examined the performance of the CPs on gender and race/ethnicity across all age groups. For the age subgroup analysis, patients were categorized based on the age at their first asthma CP-related encounter (e.g., diagnosis, prescription, or relevant keywords). Encounters prior to age 2 were excluded to ensure consistency in the age range. We used the age ranges of 2–5 years, 6–12 years, and 13–18 years. We conducted two separate analyses for the age subgroups. First, we considered all encounters the patient had in the EHR data from ages 2–18 years (with age group still defined by the age at first asthma CP-related encounter). Then, we also performed the age subgroup analysis only considering encounters within the age subgroup (e.g., for 2–5 years, only encounters when the patient was 2–5 years old.

## 3. RESULTS

The initial potential pediatric asthma cohort consisted of 128,132 patients, with 58,064 patients identified using base rules related to diagnosis and medication from structured data, while the remaining patients were identified based on asthma-related keywords from clinical notes. Manual chart reviews were conducted on 428 patients, yielding an overall agreement rate of 85.7%. Table 2 summarizes the top base rule combinations, the number of patients identified by each combination, and the number of confirmed asthma cases from manual chart reviews. The largest number of identified patients came from keyword-based identification when other rules were absent. Using diagnosis codes (Dx) at least twice alone resulted in a high proportion of confirmed asthma cases (93.8%). When keywords were incorporated, the confirmation rate increased to 94.4%. Adding inhaled corticosteroids (ICS) and keywords to the two-diagnosis rule further improved the asthma confirmation rate to 100% among reviewed patients.

We generated 416 distinct combinations of CP rules by merging the base rules with additional human-driven adjustments. For example, in some rule combinations, we removed “kw1 or kw2” due to their tendency to result in low PPV and inaccurate cases, a finding that is further supported by the chart review results presented in Table 2. The performance of those rule combinations was evaluated using several performance metrics to identify the optimal rule set that would balance the metrics, especially sensitivity, specificity, and F1-score, shown in Table 3. To achieve the highest sensitivity, the rule “dx ≥ 1 ∣ ICS ≥ 1 ∣ kw1 ≥ 1 ∣ kw2 ≥ 2” resulted in a sensitivity of 0.894 (95% CI: 0.733, 1.000). However, this rule was associated with relatively low specificity, 0.657 (95% CI: 0.450, 0.850). For the highest specificity, the rule requiring “dx ≥ 2 & ICS ≥ 1” produced the best performance, yielding a specificity of 0.981 (95% CI: 0.900, 1.000). However, this combination significantly reduced sensitivity to 0.635 (95% CI: 0.545, 0.741). When considering the F1-score, the rule “dx ≥ 1 & (ICS ≥ 1 ∣ kw1 ≥ 1)” provided the highest mean F1-score of 0.803. However, the 95% confidence interval for this rule was relatively wide (0.694, 0.905). To balance sensitivity, specificity, and F1-score, we selected the following CP rule: “dx ≥ 1 & (dx ≥ 2 ∣ IB ≥ 2 ∣ ICS ≥ 1) & (kw1 ≥ 1)”. This rule was among the top three for F1-score, with a value of 0.796 (95% CI: 0.706, 0.889), just 0.007 below the highest F1-score (0.803). It also had a relatively narrow confidence interval, and a positive predictive value (PPV) of 0.942 (95% CI: 0.800, 1.000), which was also among the top three. The sensitivity for this combination was 0.691 (95% CI: 0.586, 0.818), placing it in the top 20% of all tested rule combinations.

Our final CP, named “COMPAC’-defined as “(dx ≥ 2 ∣ dx ≥ 1 & (IB ≥ 2 ∣ ICS ≥ 1)) & kw1 ≥ 1”—successfully identified 28,878 pediatric asthma patients within the cohort. Table 4 presents the characteristics of the potential pediatric asthma cohort (extracted from the six base rules) and the final identified asthma cohorts using existing CPs (i.e., CAPriCORN, PheKB) as well as the best performing *de novo* CP COMPAC. As shown in Table 4, COMPAC, PheKB, and CAPriCORN identified similar proportions of patients across different age groups. The majority of identified patients were between ages 2–5 years, with decreasing numbers in the 6–12 and 13–18 year age groups. This pattern is expected, as the CPs define age based on the first recorded asthma diagnosis or medication use. Additionally, all CPs identified more male than female patients. Although the potential asthma cohort had a higher percentage of Non-Hispanic White (NHW) patients (48.5%), both the existing CPs and COMPAC identified more Non-Hispanic Black (NHB) patients (~ 42%) than NHW patients (~ 38%). Among co-occurring conditions identified through ICD codes, cough was the most frequently observed comorbidity, present in nearly half of the identified asthma patients. For patients identified by the CPs, various forms of albuterol were the most frequently prescribed medications.

Tables 5 and 6 present the performance comparison of the baseline CP, existing CPs, and our proposed CP (COMPAC) across demographic subgroups in the chart-reviewed cohort. Table 5 evaluates performance using all encounters before age 18 for patient identification, while Table 6 focuses on encounters restricted to the corresponding age groups. As shown in the tables, the proposed CP demonstrates the best performance by F1 score for males, non-Hispanic Whites, and the 6–12 year age group, with comparable performance for females, non-Hispanic Blacks, Hispanics, and the 13–18 year age group. In the 2–5 year age group, COMPAC had a F1 score that was 0.10 lower than the best performing CP, which was one or more diagnosis codes.

## 4. DISCUSSION

This study developed a new pediatric asthma CP, COMPAC, which utilizes a combination of diagnosis codes, BDR and ICS prescriptions, and asthma-related keywords to obtain an optimal balance of sensitivity, PPV, and F1 score. Our analysis aimed to identify the optimal combination of rules for developing a CP for asthma using multiple performance metrics. Among the tested combinations, the rule requiring at least one diagnosis code, one ICS, one kw1, or two kw2 occurrences achieved the highest sensitivity of 0.894. However, this rule led to a decrease in specificity (0.729), highlighting the common trade-off between sensitivity and specificity when aiming to capture as many true positives as possible, even at the cost of false positives. Conversely, for the highest specificity, a rule requiring at least two diagnosis codes and one ICS achieved a specificity of 0.981, but it reduced sensitivity to 0.635. This trade-off emphasizes the challenge of balancing these two metrics in phenotype development, especially in healthcare, where both false positives and false negatives can have significant consequences. In this case, lower sensitivity could result in missed asthma cases, potentially delaying interventions. The F1-score, which balances sensitivity and precision, revealed that the combination of one diagnosis code and either one ICS or one kw1 yielded the highest mean F1-score, though with a relatively wide confidence interval. This reflects the difficulty in finding a universally optimal CP rule that performs consistently across patient subsets.

To balance these competing metrics, we selected the CP rule “(dx ≥ 2 ∣ dx ≥ 1 & (IB ≥ 2 ∣ ICS ≥ 1)) & kw1 ≥ 1” as the most optimal combination. This rule ranked among the top three for F1-score, with a narrow confidence interval, a high PPV, and sensitivity in the upper quartile of all tested rules. It offers a reasonable trade-off between sensitivity, specificity, and F1-score, making it a promising candidate for asthma case identification in clinical settings. Furthermore, it incorporates both diagnosis codes and clinical features such as ICS prescriptions and asthma-related keywords, providing a more comprehensive approach to asthma identification than relying on a single data element.

In the CP development process, when identifying potential cases, the largest number of identified patients came from keyword-based identification only. However, the proportion of confirmed asthma cases by chart review in this group was relatively low, indicating that using keywords alone may not achieve high true positive rates (e.g., may identify acute upper respiratory tract infections without an underlying asthma diagnosis), while excluding them could lead to missing potential cases (e.g., multiple episodes of wheezing indicative of asthma). Adding diagnosis codes, keywords, and ICS prescriptions further improved the asthma confirmation rate to 100% among reviewed patients, highlighting the importance of combining multiple criteria for robust patient identification.

As rated by F1 score, our CP performed best for patients of male sex, non-Hispanic White patients, and in the age group of 6–12 years. Our CP exhibited comparable performance (within 0.05 F1 score) in all other subgroup categories save the 2–5 year age range. From a clinical perspective, the relative proportions of male versus female patients identified by our CP are in line with previously reported literature.^17^ However, our CP, CAPriCORN, and PheKB identified a higher proportion of Non-Hispanic Black children with asthma and a higher proportion of patients in the 2–5 year age range than prior reports. The identification of a higher proportion of Non-Hispanic Black children may be due to our underlying patient population’s demographics^18^ as compared to the United States’ national demographics.

The relatively higher identification of 2–5 year-olds may be due to the inclusion of bronchodilator prescriptions and keywords related to wheezing. That may identify more young children with asthma who are prone to wheezing with viral infections, as opposed to more strict diagnostic criteria such as pulmonary function tests or methacholine challenge tests which are rarely done in this age range. As asthma symptoms in that younger age range can be more subtle (e.g., nighttime cough) and exacerbations are more severe in younger pediatric age ranges,^19^ high sensitivity in identifying younger children with asthma is important from a clinical perspective (e.g., preventative care) and research (e.g., including more at-risk populations). However, in our age group sub-analysis, COMPAC had a F1 score of 0.761, as opposed to one or more diagnosis codes which had a best F1 score of 0.887. Therefore, there may be further work required to disentangle the clinical diagnostic challenges in this age group when examining those patients’ data in a retrospective fashion.

As for potential applications of our CP, it can be used to reduce subjectivity in clinical assessments and improve diagnostic accuracy, both in real-time clinical care and in research. The data-driven approach of a CP could be used to automate asthma case identification for clinicians and prompt periodic monitoring and preventative care. In research settings, the CP could be deployed in retrospective studies, and assist in identifying patient populations for clinical trials or observational studies, supporting further exploration of asthma care and treatment strategies.

One key area for future investigation is the validation and expansion of our CP across multiple sites to improve its generalizability. While UF Health data includes patients from both a large urban setting (Jacksonville) and a more suburban community (Gainesville), expanding the study to incorporate multiple institutions, regions, and diverse patient demographics would further strengthen the CP’s external validity. By integrating data from a broader range of healthcare settings, we can gain deeper insights into the CP’s robustness and better evaluate its applicability across different patient populations.

Another important avenue for future research is the integration of additional data sources. While our current CP relies on asthma-related signs and symptoms, diagnosis codes, and medications, future iterations could benefit from incorporating other data such as patient-reported outcomes, environmental data, or genetic factors. That expanded data could provide a more comprehensive picture of asthma in pediatric populations and improve the CP’s performance in identifying both formally diagnosed and undiagnosed cases of asthma.

### Limitations

Several limitations should be considered when interpreting the results, as outlined below. First, the analysis was based solely on data from one site, UF IDR, which may limit the generalizability of our findings. As the data come from a single institution, the developed CP may not fully capture the diversity of patient populations across other healthcare settings. Future studies should explore the applicability of this CP across multiple sites, including those with varying patient demographics and healthcare systems, to ensure broader applicability and external validity.

Second, our focus on pediatric asthma patients restricted our analysis to encounters where the patient’s age was under 18 years old. While this age limit is necessary for ensuring the phenotype is specific to pediatric asthma, it also means that we excluded patients who may have had asthma-related encounters after turning 18. In cases where patients had asthma diagnoses or related encounters after the age of 18, but did not meet the CP criteria during their pediatric years, these patients were excluded from the analysis. This exclusion may lead to a narrower scope for identifying patients with asthma who present after the traditional pediatric age range.

Third, the base rules used for developing the CPs may not be fully comprehensive. For instance, when applying the rule requiring at least two asthma-related signs and symptoms (kw1), we found that if diagnosis codes or prescriptions were also required to be absent, only 5 out of 20 chart-reviewed patients met the asthma criteria. Among these 5 patients, 1 had kw1 mentioned once, 3 had it mentioned twice, and 1 had it mentioned more than twice. Similarly, for kw2, enforcing the absence of these additional elements reduced the identified asthma cases to just one out of 20, with that patient having kw2 mentioned more than three times. These constraints make it challenging to use these rules as strict criteria for patient identification. However, omitting them entirely could lead to missing some asthma cases.

Moreover, we only extracted asthma-related signs and symptoms from clinical notes, diagnosis codes, and medications within the structured data elements of the EHR (e.g., diagnosis codes and medication prescriptions) to develop the CP. This approach may limit the phenotype’s ability to capture all potential asthma-related cases, as it may overlook other important factors such as environmental influences, patient-reported outcomes, or less structured data.

Furthermore, the evaluation of the CP performance was based on the subset of patients who underwent chart review, which might not represent the full spectrum of patients in the cohort. The selection process for chart review could introduce selection bias if certain patients were more likely to be reviewed due to their clinical characteristics or the availability of relevant data. Additionally, while we used bootstrapping techniques to estimate performance metrics, the results could still be influenced by the inherent limitations of the underlying chart data and the representativeness of the chart-reviewed sample. Lastly, while we were able to obtain and run the CAPriCORN and PheKB CPs, we were unable to obtain source code for NLP-PAC and NLP-API to compare our CPs performance to those CPs.

## 5. CONCLUSION

This study developed a CP for pediatric asthma case identification using EHR data. The CP showed promising results in identifying asthma cases, though limitations such as the reliance on a single institution’s data and the exclusion of patients with asthma-related encounters after age 18 need to be addressed in future studies. Further validation across multiple healthcare sites, integration of additional data sources, and expansion to include older pediatric patients or those transitioning to adulthood will be crucial for improving the CP’s.

## Figures and Tables

**Table 1:** Value sets, codes, and keywords used in the base CP rules.

Parameter	Value sets / codes / keywords
Asthma diagnosis codes (dx)	ICD-9-CM: 493.*; ICD-10-CM: J45.*
Medication: Inhaled Bronchodilator (BDR)	“Albuterol (albuterol, albuterol sulfate, Ventolin, Proventil, ProAir, Accuneb, VoSpire ER)”, “Ipratropium bromide (Atrovent)”, “Levalbuterol (Xopenex HFA)”, “Metaproterenol (Alupent)”, “Pirbuterol (Maxair)”, “Terbutaline (Brethine)”
Medication: Inhaled Corticosteroids (ICS)	“Beclomethasone propionate (QVAR)”, “Budesonide (Pulmicort Turbuhaler, Pulmicort Flexhaler, Pulmicort Respules)”, “Ciclesonide (Alvesco)”, “Flunisolide (Aerobid, Aerobid-M, Aerospan 80)”, “Fluticasone propionate (Flovent HFA, Flovent Diskus)”, “Mometasone (Asmanex HFA, Asmanex Twisthaler)”, “Triamcinolone acetonide (Azmacort)”
Keyword: Asthma-related signs and symptoms (kw*1)	e.g., “bronchospasm”, “bronchospastic cough”, “exercise-induced wheezing”, “exercise-induced asthma”, “asthma”, “asthma flare”, “asthma attack”, “asthma exacerbation”, “reactive airway disease (RAD)”, “asthma action plan / asthma control plan”, “pulmonology or allergy referral for asthma”, “pulmonary function tests / PFTs / spirometry confirm asthma”
Keyword: Asthma-related signs and symptoms (kw2)	e.g., “wheezing”, “dyspnea”, “shortness of breath (SOB)”, “nighttime cough”

Note: “kw*” is used to represent “keyword”.

**Table 2:** Summary of top 13 combinations of base rules, the number of patients identified by each rule combination, and the number of actual patients confirmed by manual chart reviews.

dx	BDR (≥ 2)	ICS (≥ 1)	kw1 (≥ 1)	kw2 (≥ 2)	Identified*	Covered*	Reviewed	Asthma*	Asthma / Reviewed
−	−	−	+	−	41759	105856	20	5	25.0%
−	−	−	+	+	18761	51316	20	4	20.0%
−	−	−	−	+	9548	61732	19	1	5.3%
≥ 2	+	+	+	+	8555	8555	32	30	93.8%
−	−	+	−	−	5502	28362	18	3	16.7%
≥ 2	+	−	+	+	5084	13639	23	22	95.7%
≥ 2	−	−	+	+	4727	21253	18	17	94.4%
= 1	−	−	+	−	4690	13453	21	14	66.7%
= 1	−	−	+	+	4417	7504	19	13	68.4%
= 1	−	−	−	−	4393	18855	19	10	52.6%
−	−	+	+	−	3180	21698	21	9	42.9%
≥ 2	−	+	+	+	2887	11442	20	20	100.0%
−	−	+	+	+	2502	16473	20	14	70.0%

Note: “+” indicates that the patient must meet this rule.

Identified*: “−” indicates the rule must not be met (absence).; Covered*: “−” indicates the rule is ignored.

Asthma*: Asthma cases confirmed through chart review from the selected patient sample.

**Table 3.** Performance of the baseline CP rule and rule combinations.

Rule	Sensitivity (95%CI)	PPV (95%CI)	F1-Score (95%CI)
dx≥1	0.733 (0.607, 0.889)	0.851 (0.684, 1.0)	0.785 (0.667, 0.9)
dx≥1 ∣ ICS≥1 ∣ kw1≥1 ∣ kw2≥2	0.894 (0.733, 1.0)	0.657 (0.45, 0.85)	0.752 (0.571, 0.895)
dx≥1 & (ICS≥1 ∣ kw1≥1)	0.726 (0.607, 0.864)	0.904 (0.75, 1.0)	0.803 (0.694, 0.905)
dx≥1 & (DX≥2 ∣ BDR≥2 ∣ ICS≥1 ∣ kw1≥1)	0.728 (0.607, 0.864)	0.886 (0.737, 1.0)	0.797 (0.682, 0.9)
*dx≥2	0.661 (0.567, 0.76)	0.972 (0.895, 1.0)	0.785 (0.706, 0.864)
*IB≥2 ∣ ICS≥1 ∣ kw1≥1	0.709 (0.552, 0.882)	0.689 (0.45, 0.851)	0.695 (0.514, 0.842)
*dx≥2 & ICS≥1	0.635 (0.545, 0.741)	0.981 (0.9, 1.0)	0.77 (0.696, 0.851)
*CAPriCORN [11]	0.678 (0.562, 0.8)	0.918 (0.789, 1.0)	0.778 (0.68, 0.878)
*PheKB [13]	0.62 (0.5, 0.75)	0.768 (0.579, 0.947)	0.684 (0.55, 0.8)
*(dx≥2 ∣ dx≥1 & (BDR≥2 ∣ ICS≥1)) & kw1≥1	0.682 (0.577, 0.792)	0.934 (0.8, 1.0)	0.787 (0.683, 0.87)
*dx≥2 ∣ dx≥1 & (ICS≥1 ∣ BDR≥2 & kw1≥1)	0.684 (0.586, 0.8)	0.934 (0.8, 1.0)	0.788 (0.696, 0.884)
*(dx≥2 ∣ dx≥1 & (ICS≥1 ∣ BDR≥2 & kw1≥2)	0.685 (0.581, 0.81)	0.94 (0.8, 1.0)	0.791 (0.696, 0.878)
*(dx≥2 ∣ dx≥1 & (BDR≥2 ∣ ICS≥1)) & kw1≥1	0.691 (0.586, 0.818)	0.942 (0.8, 1.0)	0.796 (0.706, 0.889)

Note: “&” represents “AND”, while “∣” represents “OR”, with “&” being evaluated first.

* Each visit can count only 1 diagnosis (dx) or 1 medication (ICS or IB) or 1 keyword (kw).

Cells highlighted in yellow indicate the highest rating of the performance metric. Green highlighting shows the CP rule with the best overall balance of performance metrics.

**Table 4:** Characteristics of the potential pediatric asthma cohort and final pediatric asthma cohorts identified by existing CPs (i.e., CAPriCORN, PheKB), and our proposed CP (i.e., COMPAC) within UFHealth IDR.

Characteristics	Potential Asthma (N = 128,132)	CAPriCORN (N = 27,827)	PheKB (N = 32,465)	COMPAC (N = 28,878)
**Age**, N (%)				
2 ~ 5	51,670 (40.3)	12,626 (45.4)	15,415 (47.5)	12,708 (44.0)
6 ~ 12	38,005 (29.7)	9,513 (34.2)	10,578 (32.6)	10,104 (35.0)
13 ~ 18	33,003 (25.8)	5,164 (18.6)	5,876 (18.1)	5,460 (18.9)
**Sex**, N (%)				
Male	67,412 (52.6)	1,5737 (56.6)	18,211 (56.1)	16,379 (56.7)
Female	60,717 (47.4)	12,089 (43.4)	14,253 (43.9)	12,498 (43.3)
Unknown	3 (0.0)	1 (0.0)	1 (0.0)	1 (0.0)
**Race/Ethnicity**, N (%)				
NHW	62,123 (48.5)	10,431 (37.5)	12,572 (38.7)	10,923 (37.8)
NHB	39,481 (30.8)	12,017 (43.2)	13,494 (41.6)	12,382 (42.9)
Hispanic	12,709 (9.9)	2,727 (9.8)	3,218 (9.9)	2,843 (9.8)
Other	4,873 (3.8)	905 (3.3)	1,086 (3.3)	933 (3.2)
**Comorbidities**, N (%)				
Cough	29,673 (23.2)	13,636 (49.0)	15,446 (47.6)	13,425 (46.5)
Fever, unspecified	27,403 (21.4)	10,150 (36.5)	11,733 (36.1)	9,945 (34.4)
Acute upper respiratory infection, unspecified	25,095 (19.6)	9,162 (32.9)	10,723 (33.0)	8,826 (30.6)
Asthma, unspecified type, unspecified	20,992 (16.4)	14,354 (51.6)	14,500 (44.7)	14,299 (49.5)
Acute upper respiratory infections of unspecified site	16,810 (13.1)	7,623 (27.4)	8,426 (26.0)	7,517 (26.0)
**Prescriptions**, N (%)				
amoxicillin (AMOXIL) 400 MG/5ML suspension	8,429 (6.6)	3,792 (13.6)	4,275 (13.2)	3,725 (12.9)
albuterol (PROVENTIL) (2.5 MG/3ML) 0.083% nebulizer solution 2.5 mg	8,054 (6.3)	5,704 (20.5)	6,127 (18.9)	5,616 (19.5)
fluticasone (FLONASE) 50 MCG/ACT Nasal Suspension	7,667 (6.0)	3,739 (13.4)	4,155 (12.8)	3,709 (12.8)
albuterol (PROAIR HFA;VENTOLIN HFA) 108 (90 BASE) MCG/ACT inhaler	7,470 (5.8)	5,539 (19.9)	5,755 (17.7)	5,566 (19.3)
amoxicillin (AMOXIL) 400 MG/5ML Suspension Reconstituted	6,980 (5.5)	2,869 (10.3)	3,348 (10.3)	2,794 (9.7)

**Table 5:** Performance comparison of the baseline CP, existing CPs, and our proposed CP (i.e., COMPAC) across demographic subgroups in the chart-reviewed cohort, using all encounters before age 18 for patient identification.

Rule	Sensitivity (95%CI)	PPV (95%CI)	F1-Score (95%CI)
**Sex: Female (N = 207)**			
dx ≥ 1	0.721 (0.583, 0.864)	0.814 (0.632, 0.95)	0.762 (0.632, 0.884)
*dx ≥ 2	0.676 (0.576, 0.8)	0.978 (0.895, 1.0)	**0.798 (0.711, 0.889)**
*CAPriCORN	0.659 (0.536, 0.783)	0.865 (0.7, 1.0)	0.746 (0.622, 0.85)
*PheKB	0.589 (0.454, 0.727)	0.704 (0.5, 0.895)	0.638 (0.488, 0.762)
COMPAC	0.689 (0.577, 0.818)	0.886 (0.737, 1.0)	0.773 (0.667, 0.87)
**Sex: Male (N = 213)**			
dx ≥ 1	0.735 (0.613, 0.864)	0.883 (0.737, 1.0)	0.8 (0.694, 0.9)
*dx ≥ 2	0.648 (0.562, 0.76)	0.961 (0.85, 1.0)	0.773 (0.692, 0.851)
*CAPriCORN	0.689 (0.581, 0.8)	0.967 (0.889, 1.0)	0.803 (0.717, 0.889)
*PheKB	0.646 (0.533, 0.769)	0.828 (0.647, 0.95)	0.723 (0.6, 0.829)
COMPAC	0.707 (0.606, 0.833)	0.979 (0.9, 1.0)	**0.82 (0.735, 0.905)**
**Race: NHW (N = = 209)**			
dx ≥ 1	0.71 (0.583, 0.857)	0.835 (0.666, 1.0)	0.765 (0.636, 0.878)
*dx ≥ 2	0.648 (0.559, 0.76)	0.973 (0.895, 1.0)	0.776 (0.696, 0.857)
*CAPriCORN	0.664 (0.562, 0.783)	0.926 (0.789, 1.0)	0.772 (0.667, 0.87)
*PheKB	0.6 (0.476, 0.739)	0.758 (0.556, 0.944)	0.668 (0.533, 0.8)
COMPAC	0.696 (0.586, 0.826)	0.96 (0.842, 1.0)	**0.805 (0.714, 0.889)**
**Race: NHB (N = 133)**			
dx ≥ 1	0.748 (0.609, 0.895)	0.829 (0.65, 0.95)	0.784 (0.65, 0.9)
*dx ≥ 2	0.68 (0.588, 0.8)	0.958 (0.85, 1.0)	**0.794 (0.708, 0.889)**
*CAPriCORN	0.678 (0.565, 0.8)	0.88 (0.737, 1.0)	0.764 (0.666, 0.864)
*PheKB	0.634 (0.5, 0.765)	0.757 (0.579, 0.947)	0.688 (0.558, 0.81)
COMPAC	0.701 (0.586, 0.842)	0.883 (0.7, 1.0)	0.78 (0.667, 0.884)
**Race: Hispanic (N = 38)**			
**Sex: Female (N = 207)**			
dx ≥ 1	0.658 (0.538, 0.779)	1 (1.0, 1.0)	**0.792 (0.7, 0.876)**
*dx ≥ 2	0.489 (0.391, 0.643)	1 (1.0, 1.0)	0.654 (0.562, 0.783)
*CAPriCORN	0.593 (0.48, 0.75)	1 (1.0, 1.0)	0.742 (0.649, 0.857)
*PheKB	0.689 (0.56, 0.833)	0.757 (0.55, 0.9)	0.718 (0.571, 0.837)
COMPAC	0.591 (0.48, 0.706)	1 (1.0, 1.0)	0.741 (0.649, 0.828)
**Age group: 2 ~ 5 (N = 173)**			
dx ≥ 1	0.893 (0.762, 1.0)	0.886 (0.722, 1.0)	**0.887 (0.769, 0.973)**
*dx ≥ 2	0.478 (0.364, 0.617)	1 (1.0, 1.0)	0.644 (0.533, 0.763)
*CAPriCORN	0.621 (0.5, 0.786)	1 (1.0, 1.0)	0.764 (0.667, 0.88)
*PheKB	0.615 (0.474, 0.778)	0.846 (0.636, 1.0)	0.709 (0.556, 0.848)
COMPAC	0.618 (0.5, 0.786)	1 (1.0, 1.0)	0.761 (0.667, 0.88)
**Age group: 6 ~ 12 (N = 132)**			
dx ≥ 1	0.711 (0.571, 0.85)	0.809 (0.632, 0.95)	0.754 (0.615, 0.865)
*dx ≥ 2	0.64 (0.556, 0.741)	0.95 (0.85, 1.0)	0.764 (0.681, 0.851)
*CAPriCORN	0.665 (0.552, 0.792)	0.888 (0.737, 1.0)	0.759 (0.651, 0.864)
*PheKB	0.569 (0.444, 0.714)	0.713 (0.5, 0.895)	0.63 (0.476, 0.762)
COMPAC	0.7 (0.586, 0.818)	0.913 (0.75, 1.0)	**0.79 (0.694, 0.884)**
**Age group: 13 ~ 18 (N = 90)**			
dx ≥ 1	0.761 (0.643, 0.9)	0.947 (0.842, 1.0)	**0.842 (0.739, 0.927)**
*dx ≥ 2	0.648 (0.559, 0.76)	0.977 (0.895, 1.0)	0.778 (0.704, 0.864)
*CAPriCORN	0.669 (0.567, 0.783)	0.945 (0.842, 1.0)	0.782 (0.694, 0.87)
*PheKB	0.612 (0.5, 0.741)	0.834 (0.65, 1.0)	0.704 (0.578, 0.818)
COMPAC	0.686 (0.586, 0.8)	0.949 (0.842, 1.0)	0.795 (0.708, 0.884)

Note: *Each visit can count only 1 diagnosis (dx) or 1 medication (ICS or BDR) or 1 keyword (kw).

**Table 6:** Performance comparison of the baseline CP, existing CPs, and our proposed CP (COMPAC) across demographic subgroups in the chart-reviewed cohort, using only encounters within the corresponding age groups for patient identification.

Rule	Sensitivity (95%CI)	PPV (95%CI)	F1-Score (95%CI)
**Age group: 2 ~ 5 (N = 173)**			
dx ≥ 1	0.597 (0.484, 0.72)	0.822 (0.632, 1.0)	0.69 (0.565, 0.8)
*dx ≥ 2	0.621 (0.54, 0.731)	0.944 (0.85, 1.0)	0.748 (0.667, 0.833)
*CAPriCORN	0.608 (0.515, 0.731)	0.896 (0.75, 1.0)	0.723 (0.615, 0.826)
*PheKB	0.542 (0.414, 0.68)	0.724 (0.526, 0.9)	0.618 (0.476, 0.75)
COMPAC	0.632 (0.533, 0.741)	0.926 (0.8, 1.0)	**0.75 (0.654, 0.833)**
**Age group: 6 ~ 12 (N = 132)**			
dx ≥ 1	0.648 (0.548, 0.76)	0.949 (0.842, 1.0)	0.768 (0.681, 0.851)
*dx ≥ 2	0.635 (0.552, 0.741)	0.98 (0.9, 1.0)	0.769 (0.702, 0.851)
*CAPriCORN	0.637 (0.556, 0.741)	0.981 (0.9, 1.0)	0.771 (0.702, 0.851)
*PheKB	0.6 (0.5, 0.708)	0.864 (0.7, 1.0)	0.707 (0.596, 0.809)
COMPAC	0.64 (0.559, 0.741)	0.985 (0.9, 1.0)	**0.775 (0.706, 0.851)**
**Age group: 13 ~ 18 (N = 90)**			
dx ≥ 1	0.594 (0.5, 0.704)	0.83 (0.65, 0.951)	0.691 (0.578, 0.792)
*dx ≥ 2	0.636 (0.556, 0.741)	1 (1.0, 1.0)	**0.777 (0.714, 0.851)**
*CAPriCORN	0.632 (0.545, 0.741)	0.972 (0.9, 1.0)	0.765 (0.691, 0.844)
*PheKB	0.593 (0.5, 0.704)	0.85 (0.7, 1.0)	0.697 (0.596, 0.8)
COMPAC	0.632 (0.556, 0.741)	0.973 (0.9, 1.0)	0.765 (0.692, 0.844)

Note: *Each visit can count only 1 diagnosis (dx) or 1 medication (ICS or BDR) or 1 keyword (kw).

## Abbreviations

BDR: bronchodilator
CP: computable phenotype
EHR: electronic health record
ICS: inhaled corticosteroid
